# Draft Genome Sequence of Nocardioides alcanivorans NGK65^T^, a Hexadecane-Degrading Bacterium

**DOI:** 10.1128/mra.01213-21

**Published:** 2022-07-18

**Authors:** Alexander Bartholomäus, Daniel Lipus, Julia Mitzscherling, Joana MacLean, Dirk Wagner

**Affiliations:** a GFZ German Research Centre for Geosciences, Section Geomicrobiology, Potsdam, Germany; b University of Potsdam, Institute of Geoscience, Potsdam, Germany; University of Southern California

## Abstract

The Gram-positive bacterium Nocardioides alcanivorans NGK65^T^ was isolated from plastic-polluted soil and cultivated on medium with polyethylene as the single carbon source. Nanopore sequencing revealed the presence of candidate enzymes for the biodegradation of polyethylene. Here, we report the draft genome of this newly described member of the terrestrial plastisphere.

## ANNOUNCEMENT

The accumulation of plastic debris in aqueous and terrestrial environments has become one of the major challenges of the 21st century and significantly contributes to a growing global pollution problem (1). While plastic debris can generally be degraded slowly by nature through photooxidative and thermooxidative depolymerization processes, the scientific focus has shifted toward the investigation of plastic-associated microorganisms with the ability to break down plastic polymers (2, 3).

Nocardioides alcanivorans NGK65^T^ is a recently described Gram-negative bacterial strain that was isolated from plastic-polluted soil from an abandoned landfill in eastern Germany (52°02′58.8″N, 12°39′34.8″E) (4). Microbial isolates were enriched in slurries with plastic debris from the landfill and minimal salt medium (MSM) containing cycloheximide (1% of a 1 M cycloheximide solution) but no additional carbon source according to a protocol described by Burd (5). After 3 to 5 days at 27°C, slurries were plated on solid MSM containing 0.1% powdered and UV-weathered polyethylene as an additional carbon source. Single colonies were transferred from polyethylene-containing MSM to MSM with 1% *n*-hexadecane as the only carbon source. In addition, growth on hexadecane as the sole carbon source was verified by monitoring the optical density (600 nm) in liquid MSM. After cultivation on 1/2x LB medium, genomic DNA of strain NGK65^T^ was extracted from cell material resuspended in extraction buffer using the UltraClean microbial DNA isolation kit (MoBio, Carlsbad, CA, USA).

High-molecular-weight DNA was prepared without specific size selection using the rapid barcoding sequencing kit (Oxford Nanopore Technologies [ONT], Oxford, UK) and was cleaned using AMPure XP beads (Beckman Coulter, Pasadena, CA). The resulting sequencing library was sequenced using the MinION platform (ONT) and the FLO-MIN106 flow cell. The sequencing ran for 72 h, and the quality was monitored using the MinKNOW interface v21.06.0 (ONT). Default parameters were used for all software unless otherwise specified. Raw sequencing data were base called and demultiplexed with high accuracy using guppy v4.4.2 + 9623c1626 (ONT), resulting in 244,068 raw reads with a median length of 3,758 bp. Assembly and polishing were performed with Flye v2.8.2-b1689 (6) (parameters: –plasmid –meta). Draft genome quality was assessed using the lineage_wf workflow, and full-length 16S rRNA sequences were recovered using the ssu_finder tool of CheckM v1.0.13 (7). Draft genome characteristics were assessed using QAST v5.0.2 (8).

The resulting draft genome had a size of 4,918,204 bp across 57 contigs, with a GC content of 68.2%, an *N*_50_ value of 125,013 bp, and coverage of 139×. The draft genome was found to be 91.7% complete and 4.5% contaminated. The taxonomic affiliation was inferred using GTDB-Tk v1.5.0 (9), which confirmed that the isolate belonged to the genus *Nocardioides*. The recovered draft genome was compared to other *Nocardioides* genomes by calculating the average nucleotide identity (ANI) using the JSpeciesWS online tool (10; https://jspecies.ribohost.com/jspeciesws/) (accessed 21 September 2021). Results suggested that the strain NGK65^T^ draft genome was most closely related to Nocardioides daejeonensis MJ31^T^ (QHKZ00000000), which was isolated from a sewage disposal plant (11) (ANIb, 78.29%; ANIm, 84.75%). A full-length 16S rRNA sequence comparison using NCBI BLAST (12) revealed 98.6% sequence identity to Nocardioides daejeonensis strain MJ31^T^ across 1,468 bp.

The annotation of the recovered draft genome using the NCBI Prokaryotic Genome Annotation Pipeline (PGAP) (13) indicated the presence of putative genes thought to be essential for the microbial breakdown of polyethylene (2, 14–16). The following putative genes were found in the reconstructed Nocardioides alcanivorans NGK65^T^ draft genome: alkane 1-monooxygenase (EC 1.14.15.3 [locus tag L1066_RS06220]) and flavin-dependent monooxygenases (EC 1.14.14 [locus tags L1066_RS12955, L1066_RS06710, and L1066_RS10445]).

### Data availability.

The draft genome of Nocardioides alcanivorans NGK65^T^ was deposited in GenBank with the accession number JAKGRW000000000 and the BioProject accession number PRJNA797358. The gene annotation is available via the accession number NZ_JAKGRW010000000. Raw reads are accessible via the accession number ERR7416542, and the 16S rRNA sequence is available via the accession number ON063448. The strain is available at the German Collection of Microorganisms and Cell Cultures (DSM 113112^T^) and the Netherlands Culture Collection of Bacteria (NCCB 100846^T^).
